# Joint Optimization of Time Slot and Power Allocation in Underwater Acoustic Communication Networks

**DOI:** 10.3390/s26072188

**Published:** 2026-04-01

**Authors:** Xuan Geng, Yongkang Hu

**Affiliations:** College of Information Engineering, Shanghai Maritime University, Shanghai 201306, China; 202330310048@stu.shmtu.edu.cn

**Keywords:** time slot allocation, power allocation, underwater acoustic communication networks (UACNs), DQN, MADDPG

## Abstract

This paper proposes a joint optimization algorithm based on reinforcement learning to address the time slot and power allocation problem in underwater acoustic communication networks (UACNs). By maximizing the total capacity of successful transmissions as the optimization objective, two sub-objectives are formulated corresponding to time-slot scheduling and power allocation. The sub-objective corresponding to time-slot scheduling is addressed by constructing a Markov Decision Process (MDP) model based on Deep Q-Network (DQN) learning. In this model, the agent learns the time slot allocation policy with the goal of increasing the number of successfully transmitted links while reducing the collision. For the sub-objective corresponding to power allocation, another MDP model is developed, solved by the Multi-Agent Deep Deterministic Policy Gradient (MADDPG) algorithm, in which each underwater transmission node acts as an independent agent. The MADDPG approach enables the system to improve channel capacity under energy limitation, which maximizes the total capacity of successfully transmitted links. In terms of model execution, the DQN adopts a centralized training and time slot allocation, while MADDPG uses a centralized training and distributed execution to select the transmission power by each node. Simulation results show that the proposed joint optimization algorithm demonstrates better performance in the number of successfully transmitted links and channel capacity compared to TDMA, Slotted ALOHA, and other algorithms.

## 1. Introduction

With increasing interest in marine exploration, researchers have increasingly focused on Underwater Acoustic Communication Networks (UACNs) [1,2,3]. Compared to terrestrial wireless communication, the underwater communication environment is much more challenging. Because of the distinctive physical properties of underwater acoustic channels, including extended propagation delays and restricted bandwidth, the transmission rate and signal quality of underwater acoustic communication networks are significantly degraded [4]. Furthermore, replacing node batteries in underwater acoustic communication networks is extremely difficult, leading to a low energy consumption requirement. Therefore, the efficient and dependable allocation of underwater communication resources under strict energy limitations has emerged as a major research focus in the field of underwater acoustic communications.

Several media access control (MAC) approaches have been widely studied, including methods based on Frequency Division Media Access (FDMA), Time Division Media Access (TDMA)-like schemes, and hybrid MAC. However, FDMA methods have limitations including limited bandwidth, multipath fading, and frequency-selective fading. While FDMA divides the available bandwidth into sub-bands, this frequency-based access leads to inefficiencies, especially as the number of nodes increases, making spectrum management more complex and reducing efficiency [5]. Although hybrid MAC protocols offer advantages in certain scenarios, they introduce additional communication overhead and greater system complexity. These drawbacks become more obvious in dynamic underwater environments, which reduce their practical applicability. In contrast, TDMA-like approaches use time division, which makes them effective in bandwidth-limited and dynamically changing underwater channels. Therefore, we choose an TDMA-like approach to optimize time-slot scheduling, thereby avoiding signal collisions, enhancing throughput [6], and reducing transmission delays [7]. M. Liu et al. [8] introduced an MAC protocol that utilizes packet-level time-slot scheduling, employing both optimal and heuristic algorithms to determine the scheduling strategy. It used a backtracking algorithm and greedy strategies to reduce computational complexity and enhance transmission efficiency. Y. Wang et al. [9] introduced a concurrent MAC protocol based on master–slave transmission.

With regard to power control, it improves performance—such as by maximizing network capacity [10] and reducing energy consumption [11]—by optimizing the transmission power of the nodes. H. Wang et al. [12] developed an algorithm for joint power allocation across multiple nodes, utilizing a hierarchical game learning approach. By constructing a multi-agent Multi-Armed Bandit (MAB) game model and employing specific learning strategies, the algorithm enhances learning efficiency and adaptability effectively. For multi-user underwater acoustic communication systems, K. Gharsalli et al. [13] introduced a power distribution technique based on non-cooperative game theory. By using an iterative water-filling algorithm and deriving the Nash equilibrium, the algorithm optimizes power allocation to maximize users’ transmission rates. However, most existing methods rely on simplified environmental models and fixed decision policies, which may be inadequate for adapting to the complexities and variations of underwater acoustic channels.

The complexity and variability of the underwater communication environment make it difficult for devices to obtain prior information. With the appearance of artificial intelligence, reinforcement learning (RL) [14] has been a potential method for addressing challenges in underwater acoustic communication networks due to its ability to learn and adapt in unknown environments. In particular, Q-learning- and deep Q-learning network (DQN)-based algorithms, have been widely applied to tasks such as time-slot scheduling [15,16] and power allocation [17,18,19]. However, since DQN is primarily designed for discrete action spaces, the agent may fail to learn optimal policies for continuous power allocation. Deep Deterministic Policy Gradient (DDPG) can generate a deterministic action through a deterministic network, making it more suitable than DQN for continuous power allocation. S. Han et al. [20] proposed a hierarchical reinforcement learning framework combining DQN and DDPG to address the joint relay selection and power allocation problem in UACNs. However, this algorithm models underwater transmitting nodes as independent agents and does not fully take into account the interference of multiple underwater transmitting nodes. In response to this issue, our previous work [21] proposed a power allocation method based on Multi-Agent Deep Deterministic Policy Gradient (MADDPG). Each transmitting node is modeled as an agent, and a centralized training with decentralized execution (CTDE) mechanism is introduced, enabling multiple transmitting nodes to learn collaboratively. This approach effectively mitigates inter-node interference and improves system performance.

Time-slot scheduling and power allocation can improve communication quality in underwater acoustic communication systems. However, the lack of coordination often limits the global optimization of network. Joint optimization of time slot and power allocation has become an effective approach to address the issues mentioned above. C. Wang et al. [22] introduced a joint algorithm for power allocation and time-slot scheduling based on quasi-interference alignment. They reformulated the original optimization problem into two smaller tasks. The first subproblem minimizes receiving time through linear programming-based time-slot scheduling, while the second subproblem maximizes throughput by adjusting signal block power via nonlinear optimization and the subgradient method. T. Zhang et al. [23] proposes a joint time-slot scheduling and power allocation algorithm based on deep multi-agent reinforcement learning (ICRL-JSA). In each time slot, each transmitting node simultaneously makes time-slot scheduling and power allocation decisions, which maximizes network throughput and maintains communication fairness. Dai et al. [24] proposed a joint optimization framework aimed at minimizing the total energy consumption in UAV-assisted computation offloading scenarios in marine networks. They developed a hierarchical joint optimization algorithm that combines an outer one-dimensional search with inner convex optimization for solution derivation. In [25], an alternating optimization framework was introduced to solve the scheduling and power allocation problems under fixed power or fixed time slot constraints. The scheduling problem was addressed using the weighted Kuhn–Munkres algorithm, while the power allocation was solved through continuous convex approximation and fractional programming iterations. Furthermore, a distributed implementation that uses a multi-leader multi-follower Stackelberg game is proposed to reduce the complexity.

Although previous research has demonstrated that joint optimization of time-slot and power allocation can enhance the performance of underwater acoustic communication networks, several limitations still exists. Traditional decomposition or alternating optimization methods often rely on strong modeling assumptions and centralized control methods. These methods are difficult to adapt to dynamic, time-varying acoustic channels. While some learning-based approaches use multi-agent mechanisms, decision-making remains largely independent for each node, with node collaboration relying heavily on rewards or indirect constraints. This approach does not effectively address the collisions causing by concurrent transmissions, which results in increased interference. Furthermore, in an end-to-end joint optimization approach, both discrete time-slot scheduling and continuous power control are learned within a single joint action space. This results in a rapid increase in the dimensionality of the action space, and the problem becomes more severe as the number of nodes grows. Therefore, it is necessary to develop a framework that can schedule nodes for concurrent transmissions while jointly optimizing the transmit power of the scheduled nodes. The framework should enable coordinated optimization among nodes while reducing the complexity of the action space, thereby improving training stability and accelerating convergence.

To address these challenges, this paper investigates the joint optimization of time-slot scheduling and power allocation in underwater acoustic communication networks and proposes a novel framework using deep reinforcement learning. The original mixed-integer nonconvex optimization problem is reformulated into two sub-objectives corresponding to time-slot scheduling and power allocation. During the time-slot scheduling, concurrent transmissions from multiple nodes within the same time slot may lead to collisions in underwater acoustic networks. To address this issue, a DQN-based approach is adopted to select the nodes that are allowed to transmit simultaneously in each time slot. This global decision process determines the time slot allocation for each node. As a result, the time-slot scheduling is made from a global perspective rather than considering nodes as independent agents, which allows better coordination among nodes. After that, power allocation is carried out for the selected nodes. To address the interference and the limited energy, a MADDPG-based power control is developed. Under a centralized training and decentralized execution framework, the transmit power of the scheduled nodes is determined through coordinated decisions. With global feedback from the shared critic network, each node learns and improves its power control policy within this framework. It should be noted that, within each time slot, the time-slot scheduling and power allocation are optimized in an iterative manner until the joint optimization of slot allocation and power allocation is achieved. The proposed method achieves an optimization of discrete decisions and continuous resource allocation, thereby improving system performance. The simulation results demonstrate that the proposed algorithm achieves a significant improvement in channel capacity and other performances compared with TDMA, slotted ALOHA, and baseline algorithms. The key contributions of this paper include the following:

1. We introduce a novel framework based on reinforcement learning for joint time slot and power optimization in underwater acoustic communication networks. Instead of regarding the optimization as a single and complex task, two sub-objectives corresponding to time-slot scheduling and power allocation are considered. These two sub-objectives are optimized in an iterative manner, allowing the system to converge to the optimal solution. The proposed design significantly reduces the complexity of the original mixed-integer optimization problem. It also enables more efficient optimization and provides better scalability as the network nodes number grows.
2. Two MDP models are designed for solving the time-slot scheduling and power allocation. The time-slot scheduling is modeled as a discrete MDP solved by DQN, while the power allocation is formulated as a continuous MDP handled by MADDPG. The MDP models introduced in this work are particularly effective for scenarios involving both discrete and continuous action spaces. They also allow additional performance metrics to be incorporated into the state and reward design.

## 2. System Model

**Figure 1:**
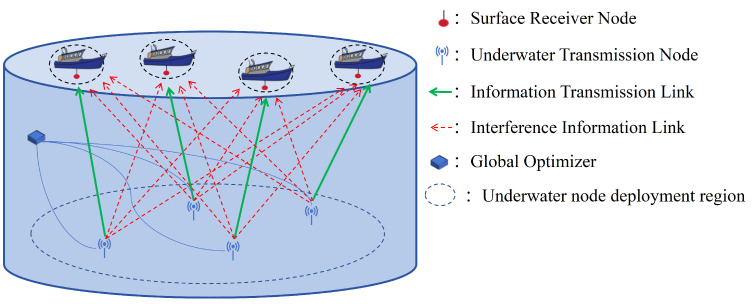
Transmission model.

The UACN model analyzed in this paper is illustrated in Figure 1, and includes transmitting nodes M={1,2,…,M} underwater, receiving nodes N={1,2,…,N} on the water’s surface, and a global optimizer. Therefore, there are a total of M×N links in the system. We assume in this paper that M=N, and each time slot lasts for $T_{s}$. When a transmission occurs from a transmitting node to its corresponding receiving node, other transmitting nodes’ signals in the same time slot are considered to be interference. When the propagation delay between the signal arrivals from the sender node and the interfering node at the receiving node is smaller than the packet duration, a collision will occur, leading to a reception failure.

At the time slot *t*, the arrival time of the signal transmitted by the transmitting node $i\left(i\in M\right)$ to the receiving node $j\left(j\in N\right)$ is given by

(1) $$\begin{array}{c} \kappa_{i,j}=T_{t}+\tau_{i,j} \end{array}$$

where $T_{t}$ is the transmission start times of the signals sent by each node at the time slot *t*. $\tau_{i,j}$ denotes the signal’s propagation delay between node *i* and node *j*, which is affected by the distance between the nodes and the acoustic properties of the underwater medium. Similarly, for the signal from the interfering node $k\left(k\in M,k\neq i\right)$ to the receiving node *j*, the arrival time is defined as

(2) $$\begin{array}{c} \kappa_{k,j}=T_{t}+\tau_{k,j} \end{array}$$

where $\tau_{k,j}$ is the signal’s propagation delay between node *k* and node *j*. If the time difference between the arrival times of $\kappa_{i,j}$ and $\kappa_{k,j}$ is smaller than the packet duration, a collision will occur. The condition is

(3) $$\begin{array}{c} |\kappa_{i,j}-\kappa_{k,j}|<T_{blk} \end{array}$$

where $T_{blk}$ represents the packet duration.

**Figure 2:**
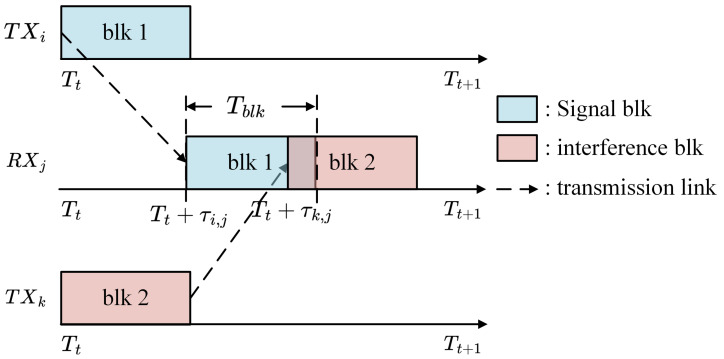
Collision timing diagram.

We define a collision function $c_{i}$, which indicates whether a collision occurs when node *i* transmits signals to node *j*. It is defined as

(4) $$\begin{array}{c} c_{i}=\left\{\begin{array}{cc} 1, & \mathrm{if}\;\left|\kappa_{i,j}-\kappa_{k,j}\right|<T_{\mathrm{blk}}\\ 0, & \mathrm{else} \end{array}\right. \end{array}$$

where the $c_{i}$ equals to 1 when a collision occurs. Otherwise, it takes the value 0. Therefore, the occurrence of collisions can be illustrated in Figure 2.

The packet duration is equal to the packet length divided by the transmission rate. In an underwater environment, the bandwidth affects the transmission rate. A larger bandwidth enables a higher data rate and thus shortens the packet duration. Receiver processing delay can also increase the packet duration. In addition, multipath delay spread may extend the effective signal propagation time, especially in complex underwater environments, leading to longer reception and decoding time.

To ensure that a packet can be fully transmitted within a single time slot, the time slot duration is defined as [23]

(5) $$\begin{array}{c} T_{slot}=T_{blk}+\tau_{max}+T_{guard} \end{array}$$

where $\tau_{max}$ denotes the maximum transmission delay of the node and $T_{guard}$ represents the guard time, which is introduced to account for any potential delays and other necessary time requirements.

At the time slot *t*, the channel gain between the node $i\left(i\in M\right)$ and the node $j\left(j\in N\right)$ is given by [26]

(6) $$\begin{array}{c} g_{i,j}^{\left(t\right)}=\frac{1}{A_{0}{\left(d_{i,j}^{\left(t\right)}\right)}^{k}{a\left(f\right)}^{d_{i,j}^{\left(t\right)}}} \end{array}$$

where $A_{0}$ denotes the normalization coefficient and *k* represents the route loss exponent. Although the path loss exponent may vary under different environmental and geometric conditions, we chose k=1.5 [18] to maintain consistency and simplify the modeling. $d_{i,j}^{\left(t\right)}$ is the Euclidean distance between the transmitting node $i(x_{i},y_{i},z_{i})$ and the receiving node $j(x_{j},y_{j},z_{j})$ at time slot *t*, which is calculated as

(7) $$\begin{array}{c} d_{i,j}=\sqrt{{\left(x_{j}-x_{i}\right)}^{2}+{\left(y_{j}-y_{i}\right)}^{2}+{\left(z_{j}-z_{i}\right)}^{2}} \end{array}$$

The a(f) is the absorption coefficient. According to the Thorp’s empirical formula, the a(f) is expressed by

(8) $$\begin{array}{c} 10\log a\left(f\right)=\frac{0.11f^{2}}{1+f^{2}}+\frac{44f^{2}}{4100+f^{2}}+\frac{2.75f^{2}}{10^{2}}+0.003 \end{array}$$

At time slot *t*, the signal-to-interference-plus-noise ratio (SINR) at the link (i,j) can be expressed by

(9) $$\begin{array}{c} SINR_{j}=\frac{{\left|g_{i,j}^{\left(t\right)}\right|}^{2}p_{i}^{\left(t\right)}q_{i}^{\left(t\right)}}{\sum_{k\in M,k\neq i}{\left|g_{k,j}^{\left(t\right)}\right|}^{2}p_{k}^{\left(t\right)}q_{k}^{\left(t\right)}+N\left(f\right)} \end{array}$$

where $g_{i,j}^{\left(t\right)}$ represents the channel gain from the transmitting node *i* to the receiving node *j*, $p_{i}^{\left(t\right)}$ is the transmission power of node *i*, and $q_{i}^{\left(t\right)}$ denotes the time slot allocation result for node *i*, taking values of 0 or 1, where 1 represents that the node transmits information in time slot *t*. N(f) represents environmental noise. The channel capacity at the receiving node *j* at time slot *t* is given by

(10) $$\begin{array}{c} C_{j}^{\left(t\right)}=\log_{2}\left(1+SINR_{j}^{\left(t\right)}\right) \end{array}$$

The total channel capacity of the underwater acoustic communication network is to be

(11) $$\begin{array}{c} C^{\left(t\right)}=\sum_{j=1}^{N}C_{j}^{\left(t\right)} \end{array}$$

Although the physical layer processes, such as modulation and decoding, are crucial to the system performance, they are not the primary focus of this study. To concentrate exclusively on the MAC layer performance, it is assumed that the physical channels are free from errors. This is made possible by employing robust forward error correction coding and equalization techniques.

## 3. Problem Formulation and MDP Model Design

### 3.1. Problem Formulation

This paper optimizes time slot and power allocation in UACNs. Efficient time slot and power allocation increases successful transmissions within each slot and enhances the channel capacity of each link, thereby maximizing the system’s global network capacity. It is possible to formulate the optimization aim to

(12) $$\begin{array}{cc} P1:\; & \max_{\begin{array}{c} q_{i}^{\left(t\right)},p_{i}^{\left(t\right)} \end{array}}\sum_{t=1}^{T}\sum_{j=1}^{N}C_{j}^{\left(t\right)}{𝓁}_{i,j}^{\left(t\right)}\\ s.t.\; & \;q_{i}^{\left(t\right)}\in \left\{0,1\right\},\;i\in M\\ \; & \;0\leq p_{i}^{\left(t\right)}\leq p_{\max},\;i\in M\\ \; & \;SINR_{j}\geq \eta_{th},\;j\in N \end{array}$$

where $q_{i}^{\left(t\right)}$ denotes the time slot allocation action for the transmitting node *i* at time slot *t*. Each node can only choose to either transmit or not transmit during each time slot. ${𝓁}_{i,j}^{\left(t\right)}=q_{i}\cdot \left(1-c_{i}\right)$ indicates whether the link (i,j) transmits information successfully or not, which takes the value 1 when the link transmits information without collision at time slot *t*; otherwise, it is 0. $p_{i}^{\left(t\right)}$ represents the power allocation action for transmitting node *i* at time slot *t*. The maximum transmission power is constrained by $p_{max}$. Furthermore, to ensure the communication quality, we introduce a minimum SINR threshold $\eta_{th}$, which ensures that information can be correctly decoded, thereby improving the reliability and quality of communication.

### 3.2. MDP Model

We addresses the optimization objective by formulating two sub-objectives, which corresponding to time-slot scheduling and power allocation, and solving them using a joint iterative optimization approach. We define each transmitting node as an individual agent and formulate two MDP models for time slot and power allocation, respectively. In each iteration, the DQN model is applied to optimize time slot allocation by selecting scheduling strategies based on the current state of each node. After determining the time slot allocation, the MADDPG network optimizes power allocation while considering nodes interactions. Through iterative alternation between these two networks, the system improves performance gradually as the agents adapt their strategies toward a near-optimal solution.

#### 3.2.1. MDP Model for Time Slot Allocation

In the sub-objective corresponding to time-slot scheduling, we need ${𝓁}_{i,j}^{\left(t\right)}=1$ to maximize the successful transmission, which is formulated by

(13) $$\begin{array}{cc} \; & \max_{\begin{array}{c} q_{i}^{\left(t\right)} \end{array}}\sum_{t=1}^{T}\sum_{j=1}^{N}{𝓁}_{i,j}^{\left(t\right)}\\ s.t.\; & \;q_{i}^{\left(t\right)}\in \left\{0,1\right\},\;i\in M \end{array}$$

However, optimizing this objective function independently may lead to a higher collision rate, which would impact the system performance and channel capacity negatively. To address this, we propose a weighted objective function that aims to both increase successful transmission success and reduced collisions, which is

(14) $$\begin{array}{cc} P2:\; & \max_{q_{i}^{\left(t\right)}}\sum_{t=1}^{T}\left(L^{\left(t\right)}-\zeta \rho^{\left(t\right)}\right)\\ s.t.\; & \;q_{i}^{\left(t\right)}\in \left\{0,1\right\},\;i\in M \end{array}$$

where $L^{\left(t\right)}$ is the quantity of links that successfully transmit during a time slot, $\rho^{\left(t\right)}$ is the collision rate, and ζ is the weight assigned to the collision rate in the objective function.

In problem P2, we design a centralized DQN method with a global optimizer to decide the time slot allocation of all nodes. The MDP model for this centralized DQN is defined as follows.

Action Space: We define the action $a_{1i}$ as the action of the transmitting node *i*, representing its transmission behavior. The action space for time slot allocation is defined as

(15) $$\begin{array}{c} A_{1}=\left\{a_{1i}=q_{i}|q_{i}\in \left\{0,1\right\}\right\} \end{array}$$

where $q_{i}=1$ data is transmitted at time slot *t*. Conversely, $q_{i}=0$ if no data is transmitted.

State Space: The state space at time slot *t* is defined as $s_{1i}^{\left(t\right)}=\left\{o_{1i}^{\left(t\right)},a_{1i}^{\left(t\right)}\right\}$, where $o_{1i}^{\left(t\right)}=\left\{c_{i}^{\left(t-1\right)},L^{\left(t-1\right)},\rho^{\left(t-1\right)}\right\}$ represents the observation at the current time slot. $c_{i}^{\left(t-1\right)}$ represents whether the data transmitted by the transmitting node *i* in the previous time slot collided. By incorporating collision results into the state space, the agent can avoid selecting time slots that repeatedly lead to collisions. $L^{\left(t-1\right)}$ represents the number of successful transmitting links at the previous time slot, and the agent uses this information to adjust its time slot allocation strategy. $\rho^{\left(t-1\right)}$ represents the system collision rate, which indicates network performance and helps the agent optimize its allocation strategy.

Reward: The optimization’s objective is to maximize the number of successfully transmitted links while minimizing the collision rate. To achieve this, we design a reward with a positive part for successful transmissions and a negative part that is proportional to the collision rate. The reward $r_{1i}^{\left(t\right)}$ is described as

(16) $$\begin{array}{c} r_{1i}^{\left(t\right)}=\zeta_{1}L^{\left(t\right)}-\zeta_{2}\rho^{\left(t\right)} \end{array}$$

where $\zeta_{1}$ and $\zeta_{2}$ are constant coefficients used to balance the weights of the number of successfully transmitted links and the collision rate.

#### 3.2.2. MDP Model for Power Allocation

After obtaining the time slot allocation for each transmitting node, power allocation is optimized to maximize the network’s capacity. We use centralized training and distributed execution for power allocation. Therefore, each transmitting node is also considered to be an agent, besides the global optimizer. The sub-objective corresponding to power allocation is then to be

(17) $$\begin{array}{cc} P3:\; & \max_{p_{i}^{\left(t\right)}}\sum_{j=1}^{N}C_{j}^{\left(t\right)}\\ s.t.\; & \;0\leq p_{i}^{\left(t\right)}\leq p_{\max}\cdot q_{i}^{\left(t\right)},\;i\in M\\ \; & \;SINR_{j}\geq \eta_{th},\;j\in N \end{array}$$

where the constraint considers whether the transmitting node *i* has transmitted data in the current time slot.

Compared to other reinforcement learning methods, MADDPG is especially appropriate for multi-agent scenarios with continuous action spaces. Therefore, we use the MADDPG to solve P3 in this paper. The MDP model is defined as follows.

Action Space: In this sub-objective, the action $a_{2i}$ of the agent *i* is the transmission power of the agent. The action space for power allocation is a continuous space. The action space for power allocation is defined as

(18) $$\begin{array}{c} A_{2}=\left\{a_{2i}=p_{i}|0\leq p_{i}\leq p_{\max}\right\} \end{array}$$

State Space: The state space in power allocation is defined as $s_{2i}^{\left(t\right)}=\left\{o_{2i}^{\left(t\right)},a_{i}^{\left(t\right)}\right\}$, where $a_{i}^{\left(t\right)}=\left\{a_{1i}^{\left(t\right)},a_{2i}^{\left(t\right)}\right\}$ represents the actions related to time slot and power allocation, and $o_{2i}^{\left(t\right)}=\left\{\phi_{i},C_{j}^{\left(t-1\right)}\right\}$ is the observation. $\phi_{i}$ represents the channel state information, including the channel gain of both the previous and current time slots, i.e., $\phi_{i}=\left\{g_{i,j}^{\left(t-1\right)},g_{i,j}^{\left(t\right)}\right\}$, which allows real-time power adjustment based on channel quality. $C_{j}^{\left(t-1\right)}$ represents the channel capacity of the previous time slot. Introducing channel capacity into the state space allows the system to evaluate the link quality of the previous time slot, thereby predicting the transmission capacity of the current time slot.

Reward: The goal of P1 is to maximize the capacity of scheduled links over the entire time domain. Therefore, we keep the term directly related to channel capacity in the reward function. The constraint ${SINR}_{j}⩾\eta_{th}$ of P1 sets a minimum SINR threshold. To make sure this condition is satisfied during reinforcement learning, we introduce a gating factor ψ in the capacity term. The channel capacity reward is included when the link satisfies ${SINR}_{j}⩾\eta_{th}$. The reward function constrains the SINR threshold in P1 so that the learning objective remains consistent with the feasibility of the constraint. In addition, we introduce a penalty term for deviation from the capacity threshold to address two undesirable behaviors. One occurs when the capacity drops below the threshold, which leads to poor service quality. The other occurs when nodes use excessive transmission power in order to obtain higher capacity, which increases energy consumption and interference during concurrent transmissions. With this penalty term, the learned policy tends to maintain the required QoS while achieving capacity with more reasonable power levels, which also improves the stability of the training process. The reward is defined as

(19) $$\begin{array}{c} r_{2i}^{\left(t\right)}=\psi C_{j}^{\left(t\right)}-{\left(C_{j}^{\left(t\right)}-\Gamma_{th}\right)}^{2} \end{array}$$

where $C_{j}^{\left(t\right)}$ represents the channel capacity of link (i,j). The second term of the equation is the square of the difference in channel capacity of (i,j) and its required minimum channel capacity $\Gamma_{th}$.

The gating factor ψ is defined as

(20) $$\begin{array}{c} \psi =\left\{\begin{array}{cc} 1, & SINR_{j}\geq \eta_{th}\\ 0, & SINR_{j}<\eta_{th} \end{array}\right. \end{array}$$

## 4. Joint Optimization for Time Slot and Power Allocation

**Figure 3:**
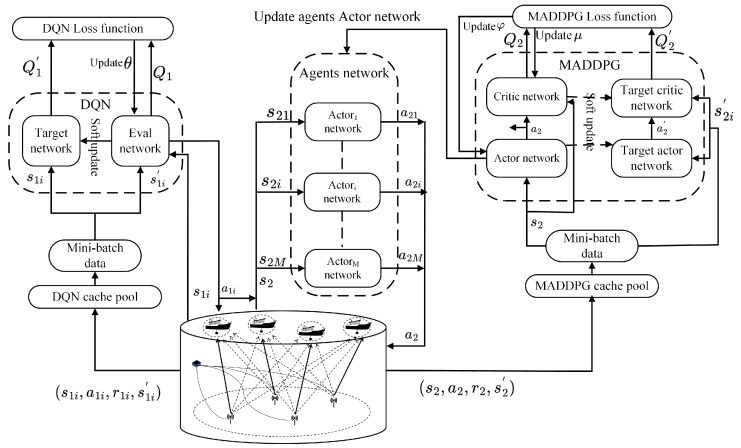
Architecture of the joint optimization algorithm.

To address the challenges of optimizing both time slot allocation and power allocation, we propose a joint optimization framework combining the DQN and the MADDPG to allocate both time slot and power, as shown in Figure 3. Time slot allocation involves a discrete action space, which is efficiently handled by DQN, a method well-suited for discrete decision-making tasks. Power allocation, on the other hand, requires the adjustment of continuous values, making MADDPG an appropriate choice due to its ability to optimize continuous action spaces through an actor–critic approach. By integrating these two algorithms into a unified framework, we enable coordinated optimization for time slot and power allocation. This hierarchical structure not only simplifies the learning process by allowing each algorithm to specialize in its respective task but also ensures more efficient and stable learning, leading to improved performance in complex multi-agent environments.

The DQN is performed using a centralized strategy with a global optimizer that includes an evaluation network and a target network. Each transmitting node gathers local state information and transmits it to the global optimizer. The optimizer then determines the optimal actions based on the global state information and then sends the decisions to the transmitting nodes for execution. For power allocation, the MADDPG adopts centralized training and distributed execution. The global optimizer contains the actor, target actor, critic, and target critic networks, and each agent maintains its own independent actor network. During training, the global optimizer updates the actor and critic networks using global information given from all agents. In the execution part, each agent selects actions independently using its local actor network based on its own observations.

**Figure 4:**
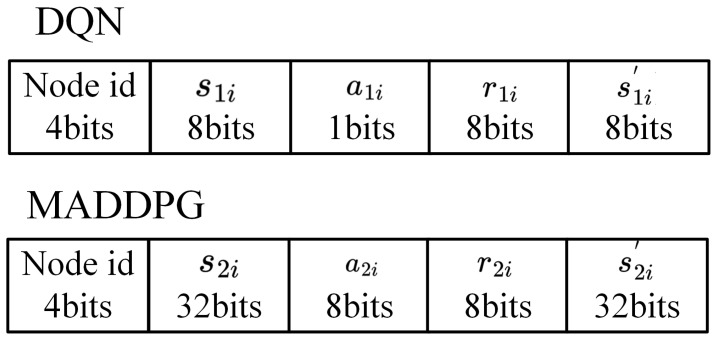
Global Optimizer Broadcast frame format.

In our framework, the global optimizer performs two roles. First, in the Centralized DQN, it serves as the global scheduler which makes slot allocation decisions based on global network information. The optimizer collects data from all nodes and broadcasts the time slot schedule decisions to each node. Once the global optimizer completes the slot allocation decision, it broadcasts the time slot schedule to each node, occurring once per second. Second, during the Centralized Training in MADDPG, the global optimizer coordinates policy updates for individual agents by gathering local information from all agents to optimize the global policy. This policy update broadcast also happens once per second, after the completion of the power allocation decision. The broadcast frame format for each node is shown in Figure 4.

Therefore, the total data size for the broadcast can be computed by N∗(84+31)∗1 bits, where *N* is the number of nodes. As the number of nodes increases, the total data size increases linearly. When the number of nodes is small, the global optimizer can efficiently manage the computational and communication load. However, as the network size grows, the broadcast burden on the global optimizer increases significantly. To address this challenge, a periodic update approach can be implemented, where the policy is updated at fixed time intervals rather than requiring a global synchronization update each time. This method can reduce the burden on the global optimizer and enhance the system’s processing capacity in larger networks.

At time slot *t*, the transmitting node *i* sends its local state $o_{1i}^{\left(t\right)}$ to the DQN network in the global optimizer. Based on this state, the global optimizer selects a time slot allocation action $a_{1i}^{\left(t\right)}$ using the ϵ-greedy strategy of the current DQN network. After all transmitting nodes execute the time slot allocation actions, the state $s_{1i}^{\left(t\right)}$ transitions to the next state s1i(t)′. Each transmitting node receives the reward $r_{1i}$ based on Equation (16), and the experience (s1i(t),a1i(t),r1i(t),s1i(t)′) is stored in the experience replay pool $E_{1}$. The global optimizer trains the DQN network by sampling mini-batches of data randomly from the $E_{1}$. Mean squared error (MSE) is used as the loss function to assess the variance between the estimated and actual network output $Q_{1}$ value and the target Q-value $Q_{1}^{\prime }$, which is

(21) $$\begin{array}{c} L\left(\theta \right)=E\left[{\left(y_{1i}-Q_{1i}\left(s_{1i},a_{1i};\theta \right)\right)}^{2}\right] \end{array}$$

where $y_{1i}$ denotes the $Q_{1i}^{\prime }$ value calculated by the DQN target network, which is

(22) $$\begin{array}{c} y_{1i}=r_{1i}+\gamma \max_{a_{1i}^{\prime }}Q_{1i}\left(s_{1i}^{\prime },a_{1i}^{\prime };\theta^{\prime }\right) \end{array}$$

where $r_{1i}$ is the reward obtained at the current time, and γ is the discount factor used to adjust the weight of future rewards. $s_{1i}^{\prime }$ and $a_{1i}^{\prime }$ represent the state and action at the next time step. $\theta^{\prime }$ is the target network weights. The DQN weights θ in the estimated network are updated by

(23) $$\begin{array}{c} \theta \leftarrow \theta -\alpha \nabla_{\theta }L_{1}\left(\theta \right) \end{array}$$

where α is the learning rate, which determines the step size for each update. The loss function’s gradient $\nabla_{\theta }L_{1}\left(\theta \right)$ represents the gradient of the loss function $L_{1}\left(\theta \right)$ with respect to θ. The parameters of the target network are periodically updated using a soft update by

(24) $$\begin{array}{c} \theta^{\prime }\leftarrow \omega \theta +\left(1-\omega \right)\theta^{\prime } \end{array}$$

where ω is an update factor, and ω≪1.

After time slot allocation, each transmitting node *i*, which we defined as agent *i* in this part, selects the power action $a_{2i}^{\left(t\right)}$ based on its local observation $o_{2i}^{\left(t\right)}$. The joint action $a_{2}^{\left(t\right)}=\left(a_{21}^{\left(t\right)},\ldots ,a_{2M}^{\left(t\right)}\right)$ is then executed by each agent. Thereafter, the global state moves to the next state s2(t)′=(s21(t)′,…,s2M(t)′). The receiving nodes feed back the reward $r_{2i}^{\left(t\right)}$ for each agent. The sample (s2(t),a2(t),r2(t),s2(t)′) is then stored in a shared replay buffer $E_{2}$. Subsequently, the global optimizer trains the MADDPG network by sampling mini-batches of data randomly from the experience replay pool $E_{2}$.

In the training process of MADDPG, mean squared error (MSE) is used as the loss function to quantify the discrepancy between the estimated network output $Q_{2i}$ and its target $Q_{2i}^{\prime }$ from the critic network, which is

(25) $$\begin{array}{c} L_{2}\left(\mu_{i}\right)=E_{\left(s_{2},a_{2},r_{2},s_{2}\right)∼E_{2}}\left[{\left(y_{2i}-Q_{2i}\left(s_{2},a_{2};\mu_{i}\right)\right)}^{2}\right] \end{array}$$

where $y_{2i}$ is the target value computed by the target critic network as

(26) $$\begin{array}{c} y_{2i}=r_{2i}+\gamma \max_{a_{2}^{\prime }}Q_{2i}\left(s_{2}^{\prime },a_{2}^{\prime };\mu_{i}^{\prime }\right) \end{array}$$

where $a_{2}^{\prime }=\left(a_{21}^{\prime },\ldots ,a_{2M}^{\prime }\right)$ is next joint action. The target actor and critic network weights The critic network parameters $\mu_{i}$ in the estimated network are updated by

(27) $$\begin{array}{c} \mu_{i}\leftarrow \mu_{i}-\alpha_{\mathrm{critic}}\nabla_{\mu_{i}}L_{2}\left(\mu_{i}\right) \end{array}$$

where $\alpha_{\mathrm{critic}}$ is the learning rate of the critic network.

The actor network $\pi_{i}\left(o_{2i},\varphi_{i}\right)$ is trained to maximize the expected return estimated by the critic. The policy gradient is computed by

(28) $$\begin{array}{c} \nabla_{\varphi_{i}}J\left(\varphi_{i}\right)=E_{s_{2}∼E_{2}}\left[\nabla_{a_{2}}Q_{2i}\left(s_{2},a_{2};\mu_{i}\right){|}_{a_{2}=\pi_{i}\left(o_{2i}\right)}\right.\\ \left.\times \nabla_{\varphi_{i}}\pi_{i}\left(o_{2i}\right)\right] \end{array}$$

the actor parameters are adjusted by ascending the gradient, which is

(29) $$\begin{array}{c} \varphi_{i}\leftarrow \varphi_{i}+\alpha_{\mathrm{actor}}\nabla_{\varphi_{i}}J\left(\varphi_{i}\right) \end{array}$$

where $\alpha_{\mathrm{actor}}$ is the learning rate of the actor network.

The target actor and critic network weights are updated gradually using a soft update technique, driven by a small factor λ

(30) $$\begin{array}{c} \mu_{i}^{\prime }\leftarrow \lambda \mu_{i}+\left(1-\lambda \right)\mu_{i}^{\prime } \end{array}$$

(31) $$\begin{array}{c} \varphi_{i}^{\prime }\leftarrow \lambda \varphi_{i}+\left(1-\lambda \right)\varphi_{i}^{\prime } \end{array}$$

where λ≪1. Each agent receives the actor network parameters from the global optimizer upon the completion of the centralized training, and each agent updates its own actor network parameters. The distribution of power is then carried out by each agency.

We conclude the joint optimization in Algorithm 1. To implement Algorithm 1 in a real-world system, a two-layer control structure combined with closed-loop training and update mechanisms could theoretically be employed. This approach is inspired by the framework outlined in [27], where the reinforcement learning module is deployed on actual underwater communication devices. In that work, the researchers used the UNETStack software along with COTS hardware, such as Raspberry Pi, USB sound cards, and transducers, to build a low-cost, software-programmable transceiver link. The reinforcement learning decision-making was embedded into the communication stack, utilizing closed-loop feedback through transmission/reception notifications, ACK/timeouts, error frames, and measurable link quality indicators for online inference and periodic updates.   
**Algorithm 1:** Joint Time Slot and Power Allocation Algorithm 1:Initialize parameters θ, $\theta^{\prime }$, μ, φ, $\mu^{\prime }$, $\varphi^{\prime }$. Experience replay pool $E_{1}$, $E_{2}$. Target network parameter update cycle $T_{u}$; 2:**for** each episode **do** 3:  Initialize the environment and state space $s_{1}$, $s_{2}$; 4:  **for** t=1 **to** *T* **do** 5:    **for** i=1 **to** *M* **do** 6:      Input state $s_{1i}$ to DQN and choose time slot allocation action $a_{1i}$ with ϵ-*greedy*; 7:      Compute reward $r_{1i}^{\left(t\right)}$ by (16); 8:      obtain next state s1i(t)′; 9:      Store experience e1i=(s1i(t),a1i(t),r1i(t),s1i(t)′) into $E_{1}$;10:    **end for**11:    Select a minibatch of samples from $E_{1}$;12:    Update DQN evaluation network θ by (23);13:    **for** i=1 **to** *M* **do**14:      Input state $s_{2i}$ to $Actor_{i}$, which outputs power allocation action $a_{2i}^{\left(t\right)}$;15:      Compute reward $r_{2i}^{\left(t\right)}$ by (19);16:      obtain next state s2i(t)′;17:      Store experience e2i=(s2i(t),a2i(t),r2i(t),s2i(t)′) into $E_{2}$;18:    **end for**19:    Select a minibatch of samples from $E_{2}$;20:    Update the Critic network parameters μ as described in (27);21:    Update the Actor network parameters φ as described in (29);22:    Broadcast $Actor_{i}$ network parameter to agent;23:    **if** $t=T_{u}$ **then**24:      Update the DQN target network parameter $\theta^{\prime }$ by (24);25:      Update MADDPG target network parameters by (30) and (31);26:    **end if**27:  **end for**28:**end for**

Based on this framework, Algorithm 1 in our paper could be implemented in a real system through a two-layer control structure combined with closed-loop training and updates. The gateway collects the network states and runs DQN to output the set of concurrently transmitting nodes for each time slot, which are then scheduled by broadcasting control frames. The scheduled nodes execute only the MADDPG actor network locally to determine transmission power, and the transmission parameters are set via the power control interface provided by the protocol stack. The receiver utilizes SINR and other link quality indicators from the protocol stack, which can serve as metadata for reception notifications or be used for ACK feedback, thus providing real-time feedback to the gateway for reward calculation and policy updates. During the training phase, the gateway receives the state, action, reward, and next state at each time step, and then store these information in the experience replay pool. Periodically, DQN is trained in small batches. The parameters of the target network are softly updated to gradually match the parameters of the current network. In the MADDPG centralized training framework, the critic and actor networks are updated, and the updated actor parameters are then sent to the underwater nodes. This procedure enables lightweight online execution and stable learning updates, even in bandwidth- and computationally constrained underwater terminal environments.

## 5. Simulation Results

**Figure 5:**
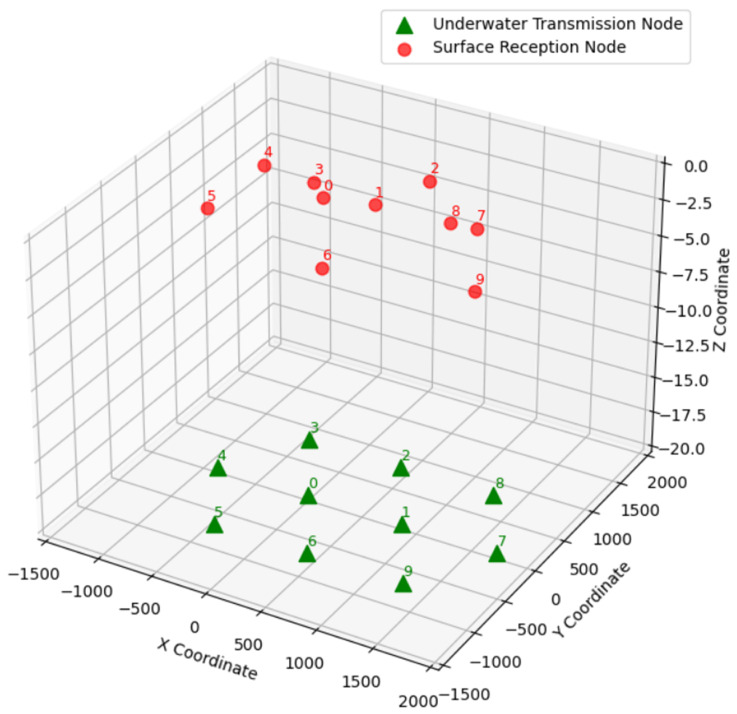
Underwater acoustic communication network node distribution model.

The simulation results of the proposed joint optimization approach, denoted by DQN-MADDPG, are presented in the following section. The simulation model for the underwater acoustic communication network is shown in Figure 5. There are 10 transmitting nodes and 10 receiving nodes, respectively. The depth of the transmitting nodes is 20 m. The transmitting nodes are uniformly distributed underwater, while the receiving nodes are randomly distributed on the surface. Each underwater transmission node has an initial energy of 5000 J and a maximum transmission power of 5 W. To ensure the statistical reliability of the results, Monte Carlo simulations are employed in this section. Each episode consists of 12,500 runs, and the results are averaged across all runs.

### 5.1. Hyperparameter Comparison

In this section, we explore the impact of different hyperparameters on the average channel capacity of our reinforcement learning algorithms. We experimented with three different sets of hyperparameters, denoted as Low, Medium, and High. The values of the hyperparameters for sets Low, Medium, and High are shown in Table 1.

**Figure 6:**
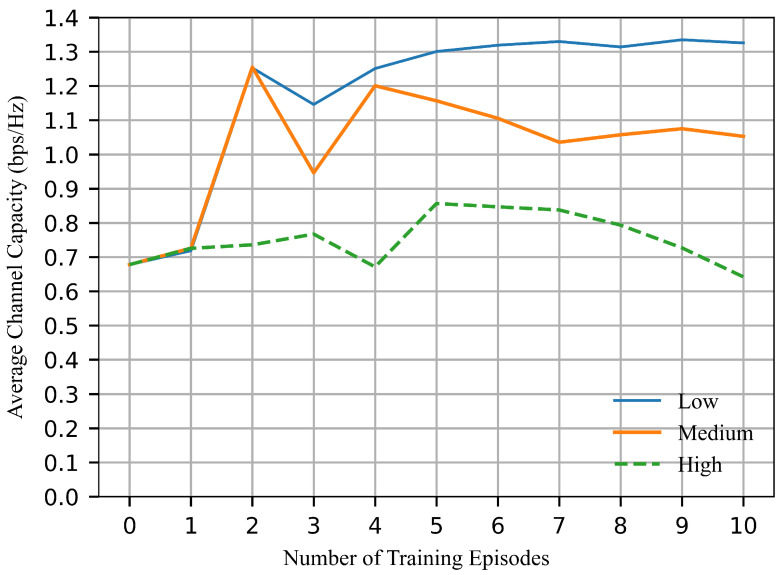
Average channel capacity with different hyperparameters.

As illustrated in Figure 6, the results are influenced by the learning policy, which leads to discontinuity during the initial phase of training. Under the “Low” learning rate, the results exhibit instability during the first three episodes and become stable after the fourth episode. It demonstrates a relatively smaller variations and faster convergence. In contrast, increasing the learning rate does not lead to further performance improvement. Instead, it results in larger variation during training. These observations indicate that a smaller learning rate facilitates a more stable and predictable learning process. Therefore, simulations in Section 5.2 to Section 5.4 use “Low” learning rate to ensure stable training, and all the hyperarameters are shown in Table 2.

### 5.2. Ablation Study

In this section, we have conducted an ablation study to evaluate the effectiveness of different modules in our approach. Specifically, we compared our method with the baseline algorithm TDMA, as well as with the individual components, which are DQN-only and MADDPG-only. Table 3 presents the results of the ablation study. The average channel capacity using the TDMA algorithm is relatively low. However, when time slot allocation based on DQN and power allocation based on MADDPG are introduced, a significant improvement in average channel capacity is observed. The results show the effectiveness of dynamically optimizing time-slot scheduling or power allocation in improving system performance. In addition, the joint optimization of time slot and power allocation maximizes the number of successfully transmitted links and the channel capacity, which demonstrates that it plays an important role in improving system performance.

### 5.3. Impact of Network Size on System Performance

**Figure 7:**
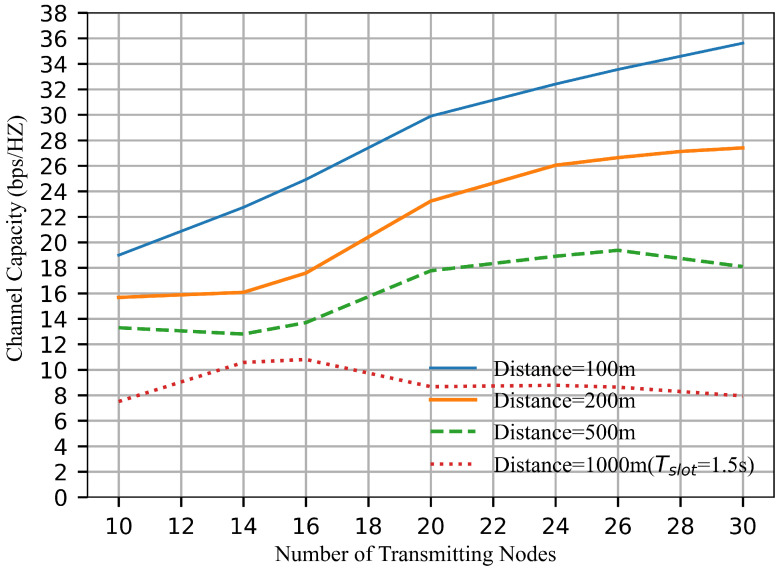
Channel capacity at different distances for varying numbers of transmitting nodes.

This section evaluates the scalability of the system through simulations involving varying node counts and inter-node distances. To ensure complete information transmission within a time slot, the slot duration is set to 1 s for node distances of 100 m, 200 m, and 500 m, while the slot duration is increased to 1.5 s for a 1000 m distance. Figure 7 shows that when the distance is 100 m or 200 m, the channel capacity increases as the number of nodes increases, which demonstrates the strong scalability of the system. The results indicate that the system can support a larger number of nodes without performance degradation. However, when the distance between nodes increases to 500 m or 1000 m, the scalability of the system begins to decrease. Although the addition of more nodes creates more opportunities for concurrent transmissions, signal attenuation and interference limit the performance in these long-distance scenarios. As a result, the growth in channel capacity slows down, and the system may reach a stage where further network growth produces little improvement or even leads to performance degradation.

### 5.4. Comparison with Baseline Methods

To evaluate the performance of the proposed joint optimization algorithm, we use MADDPG [20], R-MADDPG which introduces random slot allocation based on MADDPG, and also the ICRL-JSA algorithm from [22] as baseline algorithms. Additionally, TDMA and Slotted ALOHA algorithms, which are traditional communication algorithms, are included for comparison.

**Figure 8:**
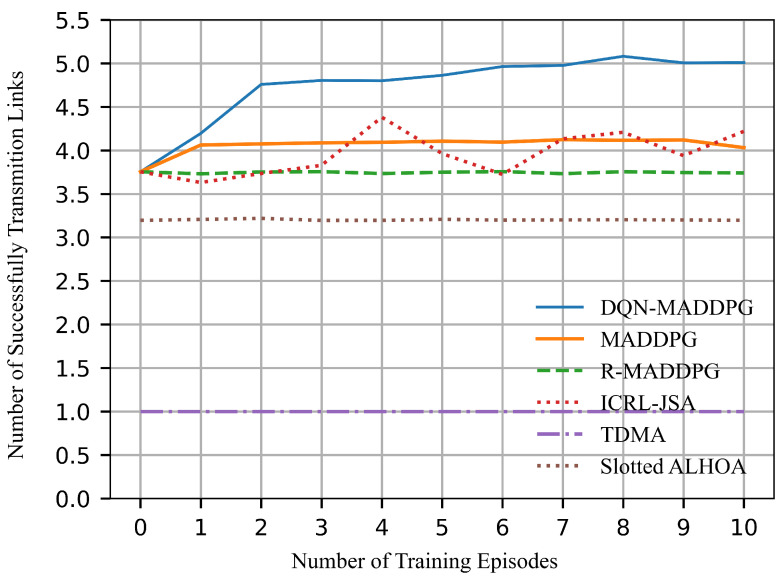
Successfully transmitted links in each time slot.

Figure 8 shows a comparison of the number of successfully transmitted links in each time slot. The results of the experiment show that our DQN-MADDPG algorithm exhibits the best performance. In our algorithm, the number of successfully transmitted links per time slot steadily increases with the number of training episodes, eventually reaching 5. In contrast, traditional algorithms such as TDMA and Slotted ALOHA demonstrate poorer performance, indicating their limited adaptability in multi-agent environments. On the other hand, algorithms like ICRL-JSA, MADDPG, and R-MADDPG yield relatively better results, but their performance is still lower than that of DQN-MADDPG. This is primarily due to MADDPG’s lack of time-slot scheduling, which prevents it from avoiding collisions. R-MADDPG, which selects transmission nodes randomly, suffers from suboptimal performance. Meanwhile, the ICRL-JSA method, which is an end-to-end joint optimization, tends to produce less stable results and provides limited optimization for single time-slot scheduling. Therefore, our method achieves the best performance.

**Figure 9:**
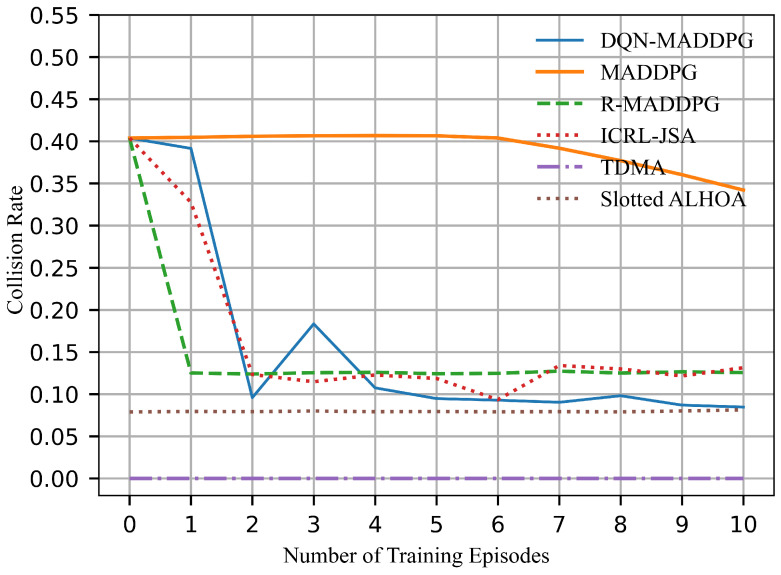
Collision rate.

Figure 9 illustrates the collision rate of the network throughout the training process. TDMA and Slotted ALOHA maintain relatively low collision rates by limiting the number of nodes selected for transmission in each time slot. In contrast, MADDPG, which does not involve time slot allocation, exhibits a higher collision rate. R-MADDPG stabilizes the collision rate around 12.5%. Although ICRL-JSA can optimize the network, its end-to-end joint optimization approach does not specifically focus on time slot allocation. This limits its ability to reduce collisions, therefore its collision rate remains around 12.5%. In contrast, the DQN-MADDPG algorithm, with its specialized optimization for time slot allocation, can reduce data packet collisions effectively. As a result, the collision rate drops below 10%, which improves the reliability of signal transmission in underwater acoustic communication networks.

**Figure 10:**
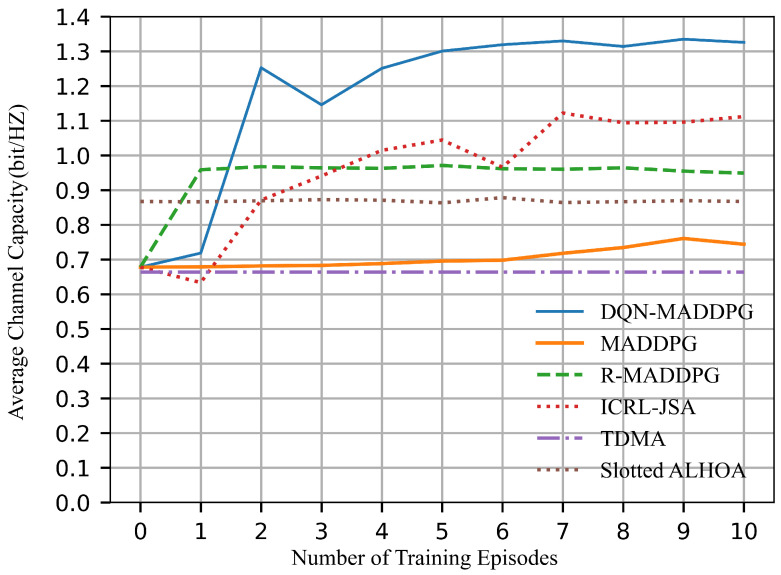
Average channel capacity.

Figure 10 shows that the DQN-MADDPG algorithm achieves the highest average channel capacity to over 1.3 bps/Hz, demonstrating its effectiveness in optimizing network performance. In contrast, TDMA, with its fixed time slot allocation for each node, minimizes interference by allowing only one node to transmit per time slot. However, it has the lowest capacity due to its limited time resources. MADDPG suffers from the most interference and consequently achieves lower capacity. Slotted ALOHA and R-MADDPG achieve capacities of around 0.87 bps/Hz and 0.96 bps/Hz, respectively. Although both methods outperform MADDPG, their capacities are still lower than those achieved by ICRL-JSA and DQN-MADDPG. ICRL-JSA achieves a capacity of 1.1 bps/Hz, but its performance remains inferior to that of DQN-MADDPG due to its limited optimization for time slot allocation and the discrete power allocation approach, which prevents it from reaching the optimal solution.

**Figure 11:**
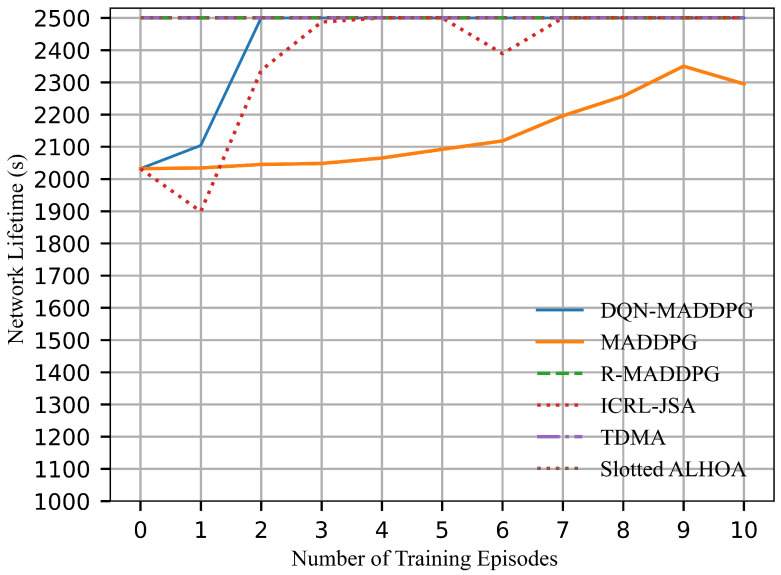
Network lifetime.

Figure 11 compares the network lifetime of the algorithms, defined as the time a node operates before its energy is exhausted within 2500 time slots. Under the same initial energy of 5000 J, the DQN-MADDPG algorithm obtains a network lifetime of 2500 s and reaches the steady-state earlier than the ICRL-JSA algorithm. Similarly, TDMA, Slotted ALOHA, and R-MADDPG also achieve a network lifetime of 2500 s by selecting fewer transmitting nodes per time slot. MADDPG maintains a network lifetime of only 2300 s, exhibiting the lowest efficiency among all the algorithms. The results demonstrate that the proposed DQN-MADDPG algorithm enhances network lifetime by allocating both energy and time resources efficiently. This enables the algorithm to achieve a balance between reducing collisions and optimizing resource utilization, thus extending the network lifetime.

**Figure 12:**
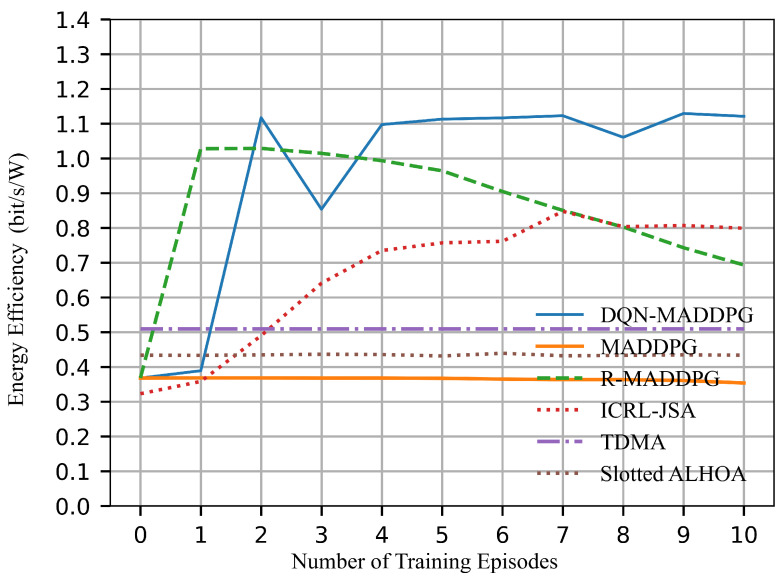
Energy efficiency.

Figure 12 illustrates the energy efficiency over training episodes for various algorithms. TDMA, Slotted ALOHA, and MADDPG exhibit relatively poor performance. Although R-MADDPG initially demonstrates comparatively high energy efficiency, its performance gradually decreases over time. ICRL-JSA converges to approximately 0.8 bits/s/W, which indicates a moderate improvement in performance. However, it still does not reach the performance achieved by DQN-MADDPG. In comparison, DQN-MADDPG maintains an energy efficiency above 1.1 bits/s/W, because it reduces collisions in time slot allocation and allocates transmission power more effectively. For the DQN-MADDPG algorithm, the temporary increase in collisions around episode 3 causes some links to consume transmission power but contribute no channel capacity. As a result, the overall energy efficiency temporarily decreases. As training continues, the energy efficiency gradually recovers and converges. By joint optimization, DQN-MADDPG achieves higher energy efficiency while maintaining good network performance.

It is worth noting that the exploration of training processes will lead to unstable results during the first few episodes. In time-slot scheduling, the collision rate shown in Figure 9 exhibits dip/discontinuity during the first three episodes. A higher collision rate implies that more nodes are scheduled to transmit simultaneously, which results in increased interference. This reduces both channel capacity and energy efficiency. Accordingly, the channel capacity in Figure 10 and the energy efficiency in Figure 12 decrease under higher collision rates during the first three episodes. However, the collision rate is not directly correlated with the number of successfully transmitted links. Therefore, the successfully transmitted links shown in Figure 8 remain relatively stable without significant discontinuity. Moreover, the network lifetime is influenced by both time-slot scheduling and power control, and thus the performance curve in Figure 11 does not exhibit evident variation with respect to changes in the collision rate. As training continues, all curves converge to stable values.

## 6. Conclusions

This paper proposes a DRL-based resource allocation method for multi-node underwater acoustic communication networks, aiming to jointly optimize time-slot scheduling and power allocation. Facing significant interference inherent in underwater communications, the proposed framework formulates two sub-objectives corresponding to time-slot scheduling and power allocation to enhance the total network capacity for successful transmissions. Two MDP models are developed for each sub-objective, enabling joint iterative optimization through a combination of DQN and MADDPG to solve the optimization goal. Simulation results show the effectiveness of the proposed DQN-MADDPG approach in complex underwater environments. It also significantly improves channel capacity and has advantages in successful transmissions, collision rate, and network lifetime compared to benchmark algorithms. Future studies will concentrate on implementing the proposed joint optimization algorithm in real-world settings to further improve its practical applicability and reliability. We will construct an experimental platform to validate the model under realistic underwater environment. This platform is supposed to provide a more accurate representation of underwater propagation characteristics and support performance evaluation beyond simulation-based analysis. In addition, we intend to deploy the proposed approach in actual underwater network environments to further evaluate performance and improve the model. Through these experimental evaluations, we aim to enhance the reliability of the model in practical applications and further optimize the design of underwater acoustic networks.

## Figures and Tables

**Table 1:** Hyperparameter values for sets Low, Medium, and High.

Parameter	Low	Medium	High
DQN Network Learning Rate	0.0005	0.005	0.05
Actor Network Learning Rate	0.0005	0.005	0.05
Critic Network Learning Rate	0.001	0.01	0.1

**Table 2:** Hyperparameters.

Parameters	Value
DQN Network Learning Rate	0.0005
Actor Network Learning Rate	0.0005
Critic Network Learning Rate	0.001
Discount Factor (γ)	0.01
episode	10
Time slot per Episode	25,000

**Table 3:** Performance comparison of ablation study results.

Methods	DQN	MADDPG	Number of Successfully Transmission Links	Average Channel Capacity (bps/HZ)
Baseline (TDMA)			1	0.6640
Time slot allocation	✓		4.6128	0.7440
Power allocation		✓	4.0314	0.7983
ours	✓	✓	5.0098	1.3258

Note: the checkmark indicates that the module is included in the simulation.

## Data Availability

The data that support the findings of this study are available from the corresponding author upon reasonable request.
